# The impact of educational interventions on COVID-19 and vaccination attitudes among patients in Michigan: A prospective study

**DOI:** 10.3389/fpubh.2023.1144659

**Published:** 2023-04-03

**Authors:** Maya Asami Takagi, Samantha Hess, Zachary Smith, Karissa Gawronski, Ayushi Kumar, Jacob Horsley, Nicholas Haddad, Bernard Noveloso, Stephen Zyzanski, Neli Ragina

**Affiliations:** ^1^Central Michigan University, College of Medicine, Mt. Pleasant, MI, United States; ^2^Central Michigan University Medical Education Partners, Saginaw, MI, United States; ^3^Department of Family Medicine and Community Health, School of Medicine, Case Western Reserve University, Saginaw, MI, United States

**Keywords:** COVID-19 virus, COVID-19 vaccines, vaccine hesitancy, educational intervention, COVID-19 attitudes, COVID-19 knowledge

## Abstract

**Background:**

Mass vaccination serves as an effective strategy to combat the COVID-19 pandemic. Vaccine hesitancy is a recognized impediment to achieving a vaccination rate necessary to protect communities. However, solutions and interventions to address this issue are limited by a lack of prior research.

**Methods:**

Over 200 patients from 18 Michigan counties participated in this study. Each participant received an initial survey, including demographical questions and knowledge and opinion questions regarding COVID-19 and vaccines. Participants were randomly assigned an educational intervention in either video or infographic format. Patients received a post-survey to assess changes in knowledge and attitudes. Paired sample *t*-tests and ANOVA were used to measure the effectiveness of the educational interventions. Participants also elected to complete a 3-month follow-up survey.

**Results:**

Patients showed increased knowledge after the educational intervention in six out of seven COVID-19 topics (*p < 0.005*). There was increased vaccine acceptance after the intervention but no difference in the effectiveness between the two intervention modalities. Post-intervention, more patients believed in CDC recommendations (*p = 0.005*), trusted the vaccine (*p = 0.001*), believed the vaccines had adequate testing (*p = 0.019*), recognized prior mistreatment in the medical care system (*p = 0.005*), agreed that a source they trust told them to receive a vaccine (*p = 0.015*), and were worried about taking time off of work to get a vaccine (*p = 0.023*). Additionally, post-intervention, patients were less concerned about mild reactions of the virus (*p = 0.005*), the rapid development of the vaccines (*p < 0.001*), and vaccine side effects (*p = 0.031*). Data demonstrated that attitude and knowledge improved when comparing pre-educational intervention to follow-up but decreased from post-intervention to follow-up.

**Conclusion:**

The findings illustrate that educational interventions improved COVID-19 and vaccine knowledge among patients and that the knowledge was retained. Educational interventions serve as powerful tools to increase knowledge within communities and address negative views on vaccination. Interventions should be continually utilized to reinforce information within communities to improve vaccination rates.

## 1. Introduction

First identified in January of 2020, severe acute respiratory syndrome coronavirus-2 (SARS-CoV-2) has resulted in significant morbidity and mortality, while also disrupting societies and economies on a global scale (1). Since being declared a Public Health Emergency of International Concern by the World Health Organization (WHO) on January 30, 2020, the disease caused by SARS-CoV-2, known as coronavirus disease 2019 (COVID-19), gained worldwide attention, and led to a unified effort to understand and treat this novel disease. Throughout 2020 the number of COVID-19 infections increased and on March 11, 2020, the WHO declared COVID-19 a pandemic (2). As the pandemic continued, vaccination against SARS-CoV-2 emerged as the most promising method of protection against COVID-19 infection (3). By late November of 2020, several pharmaceutical companies announced encouraging early results of their large-scale vaccine trials (4). Subsequently, the vaccines received Emergency Use Authorizations from the United States Food and Drug Administration and vaccine administration began as early as December 2020.

Over the months following authorization, the vaccine became widely available throughout the United States. There was a large public health initiative from private equity and national, state, and local governments to vaccinate as many individuals as possible to achieve herd immunity against the virus. While herd immunity was theoretically possible through natural infection, early predictions required a natural infection threshold of 67% to convey immunity (5). Due to the morbidity and mortality of the virus, there was a sense of urgency to curb the spread of disease through vaccination. However, this sense of urgency brought to light an issue that had been previously reported but was not fully acknowledged: vaccine hesitancy. Vaccine hesitancy has been prevalent in the United States for years, an issue that gained media attention during the 2009 influenza H1N1 outbreaks (6). Vaccine hesitancy has been such a pervasive issue that the WHO EURO Vaccine Communications Working Group developed the “5 Cs” model in 2021 to better understand the problem (7, 8). This model identifies five categories of vaccine hesitancy: confidence, complacency, convenience, communication, and context. Confidence is defined as trust in (i) the effectiveness and safety of vaccines; (ii) the system that delivers them, including the reliability and competence of the health services and health professionals; and (iii) the motivations of policymakers who decide on the needed vaccines. Complacency is defined as the perceived risks of vaccine-preventable diseases being low or that the vaccine is not deemed a necessary preventive measure. Convenience is defined as the physical availability, affordability, geographical accessibility, ability to understand (language and health literacy), and appeal of immunization services. Communication is defined as sources of information such as social media and the government, addressing and monitoring misinformation, and engaging in the benefits of the vaccination with the community. Context is defined as the consideration of ethnicity, religion, occupation, and socioeconomic status and utilizing socio-demographic characteristics in targeted campaigns (9, 10). While the issues addressed in the “5 Cs” model are of legitimate concern, they are also areas often exploited by anti-vaccination campaigns.

During the COVID-19 pandemic, anti-vaccination activists flooded social media with messages that downplayed COVID-19, questioned the truthfulness of vaccine trials, and in some cases denied the existence of COVID-19 altogether (11). Additionally, the accelerated pace of vaccine development and novel mRNA delivery system further exacerbated public anxieties regarding the vaccine (12, 13). A 2020 assessment in the United States showed that only 52% of respondents were very likely to get the COVID-19 vaccine, emphasizing the importance of implementing different strategies of intervention to promote mass immunizations (14). However, prior to attempting intervention, it is necessary to understand the factors that drive hesitancy in the first place.

The Health Belief Model (HBM) serves as a paradigm in public health to guide the promotion of health and disease prevention. This model is used to explain and predict individual changes in behaviors related to health promotion, such as perceived susceptibility, severity, benefits, and barriers (15). Components of the HBM have been utilized in previous public health interventions, such as influenza vaccination uptakes, to identify predictors for individual behaviors (16, 17). A recent systematic review found that HBM is useful in predicting COVID-19 vaccine hesitancy with the most common modifying factor being gender, followed by education, age, geographical location, occupation, income, employment, marital status, race, and ethnicity (18). While the HBM does identify variables impacting hesitancy, the best interventions to mitigate vaccine hesitancy are limited by a lack of prior research (19).

Therefore, exploring the impact of different educational interventions on COVID-19 vaccine hesitancy is essential to increase not only vaccination rates but also our understanding of how the HBM fits into COVID-19 vaccine hesitancy. Kaim et al. demonstrated the benefits of a videographic educational program on improving attitudes toward vaccination acceptability (20). However, it was noted that the study did not contain a longitudinal component, and therefore, opinions regarding the vaccine may change over time. In this study, we conduct a comparison of different modes of educational intervention (infographic vs. videographic) to assess their effectiveness in population subgroups that are initially hesitant toward vaccines. We also include a longitudinal component to examine whether vaccine acceptance changes over time. Data from this study were gathered in the state of Michigan in the United States. As different modalities of educational interventions are applicable to many public health issues, this study has great significance in guiding interventions to future pandemics or other public health emergencies.

## 2. Methods

### 2.1. Study design

A prospective study collected data using questionnaires at outpatient primary care clinics in Michigan. This study was conducted from July 2021 to July 2022. Research assistants recruited patients at outpatient waiting rooms to complete the questionnaires. This study utilized two questionnaires to understand participants’ perceived knowledge and attitude regarding SARS-CoV-2 and the COVID-19 vaccines. After informed consent was obtained, participants completed the first set of questionnaires using a pre-loaded survey on a project-issued iPad or could complete the study on their personal Smartphone device *via* a QR code. After completing the first set of surveys, participants had the option to complete a 3-month follow-up questionnaire at home to determine if their attitudes and knowledge had changed. If they chose to complete this second questionnaire, the patient’s email address was recorded and was paired to an anonymous identification number that a participant created when they completed survey. The email address was recorded *via* a secure document that was separate from the survey. This study analyzed participants’ attitudes and knowledge regarding COVID-19 and vaccine to draw conclusions about the effectiveness of the interventions. Data recorded from this study remained anonymous and the separate secure document with participants’ email addresses was the only patient-identifying information gathered during this study. Questionnaires and educational interventions were distributed by CITI-trained Central Michigan University (CMU) College of Medicine students. The CMU College of Medicine Research Institutional Review Board (IRB), Covenant Medical Center IRB, and Saint Joseph Mercy Health System and Trinity Health System Level Research IRB provided approval and oversite to maintain ethical standards and participant anonymity. Before data collection, written consent to conduct the study was obtained from community affiliations partnered with CMU College of Medicine where questionnaires were administered.

### 2.2. Participants

Participants were recruited in outpatient clinics in four counties throughout Michigan: Isabella, Saginaw, Sanilac, and Wayne. This included one clinic in Isabella County, three clinics in Saginaw County, two clinics in Sanilac County, and one clinic in Wayne County. The inclusion criteria were defined as patients at one of the previously mentioned clinics who were above the age of 18 and able to understand English. The exclusion criteria were defined as individuals who were not patients at one of the previously mentioned clinics or those who were not above the age of 18 or were unable to understand English.

### 2.3. Questionnaires and educational interventions

**Figure 1:**
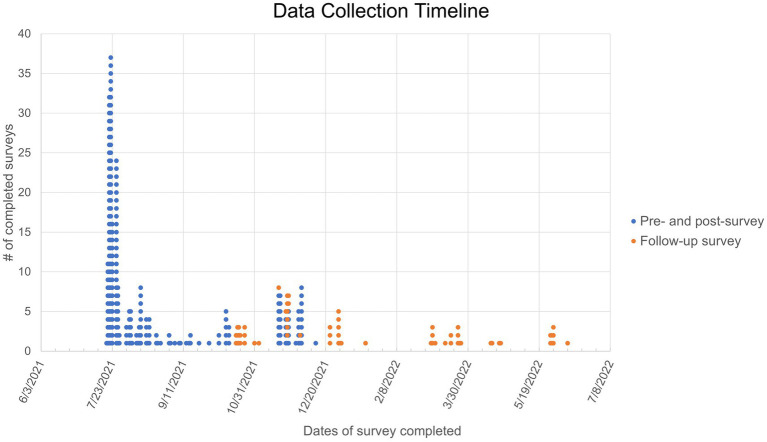
Dot plot representing survey completion dates. Each dot represents one participant. The pre- and post-survey completion dates are indicated in blue. The follow-up survey completion dates are indicated in orange.

**Figure 2:**
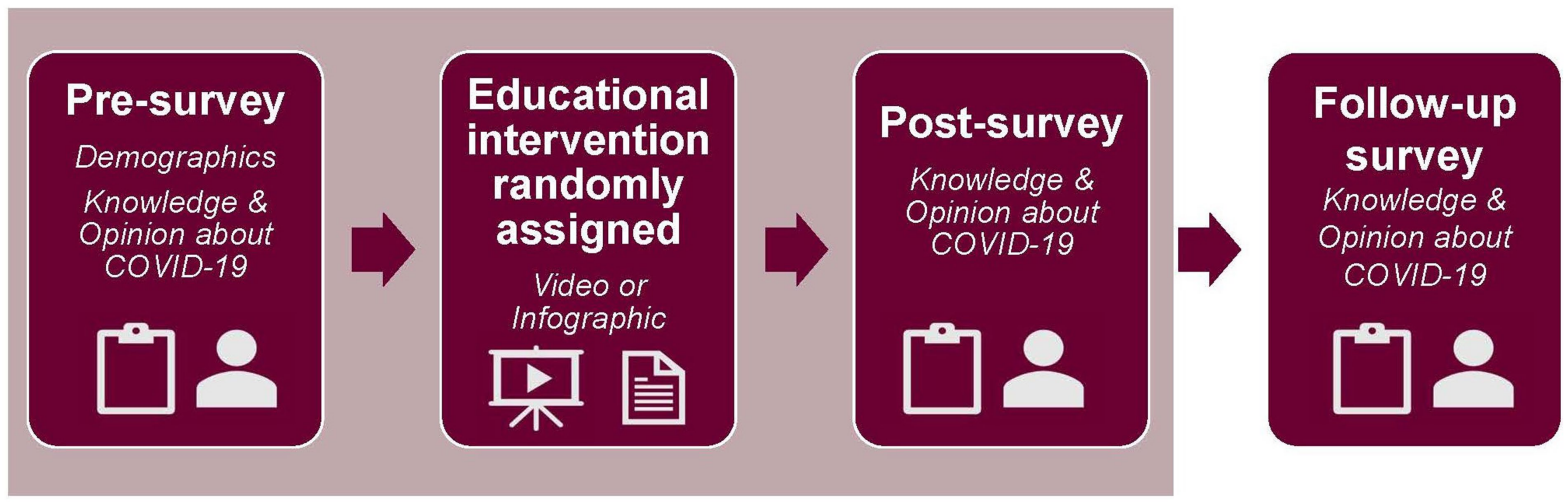
Survey flow. Three parts of the survey, illustrated under the tan-colored rectangle, were completed during one sitting. First, each participant completed the pre-survey, which included demographic and COVID-19 questions. Next, the participants were randomized to receive either a video or infographic educational intervention. Finally, the participant completed the post-survey, which included COVID-19 questions. Three months later, the participants received a follow-up survey, which included COVID-19 questions.

Two sets of anonymous surveys were distributed in this study. The first set of surveys (pre-survey and post-survey) were collected *via* Qualtrics Online Survey Platform between July 20, 2021 and December 3, 2021. The follow-up survey was collected *via* Qualtrics between October 18, 2021 and June 8, 2022 (Figure 1). The first set of surveys included a pre-survey, educational intervention, and post-survey (Figure 2). These three components were completed in one sitting. The 67-item pre-survey obtained information on the following domains: demographics, COVID-19 and vaccine knowledge, COVID-19 vaccination status, and vaccine beliefs and concerns. Questions regarding demographics, virus and vaccine knowledge, and vaccination status consisted of multiple-choice answers. Demographic data was optional to complete. Of the 67-item questionnaire, 29 of these utilized a three-point Likert scale which included 2 = agree, 1 = unsure, or 0 = disagree to assess attitude regarding COVID-19 and its vaccine. There were seven multiple choice questions aimed at assessing COVID-19 and vaccine knowledge. Next, using the Qualtrics randomization function, participants received either a seven-minute COVID-19 or vaccine educational video or received a COVID-19 and vaccine educational infographic. If participants received the infographic, they were required to spend at least four-minutes reading it before moving on to the next step (Supplementary material 1). Both materials were produced using information from the Centers for Disease Control and Prevention (CDC) and the World Health Organization (WHO) (21, 22). Immediately following the educational intervention, participants received the same COVID-19 knowledge and attitude questions they had answered in the pre-intervention questionnaire. The questions regarding demographics from the pre-intervention survey were not included in the post-intervention survey. After completion of the post-intervention questionnaire, participants received a $20 gift card for their completion of these three components.

The second survey set included a 3-month follow-up survey. After completion of the first survey, patients were offered to complete the second survey, which could be completed at home. Those that elected to complete this portion of the study created a unique identification number to maintain anonymity and to match the first set of surveys with their 3-month follow-up survey. Emails were collected from patients who were interested in completing the follow-up survey. To incentivize completion of the follow-up survey, participants received a $10 digital gift card sent to their email upon completion of this survey. The follow-up survey was distributed to patients at least 3 months after the completion of the first set of surveys. The follow-up survey prompted participants to provide updated vaccination status, provide any learning about COVID-19 or the vaccine that may have occurred since the educational intervention, and included the same COVID-19 knowledge and attitude questions from the pre- and post-intervention surveys. If answered “YES” to the question asking about new information learned since the educational intervention, a drop-down area for participants to type what they had learned appeared. Otherwise, all other questions consisted of multiple-choice questions and the three-point Likert scale questions previously described. This questionnaire also utilized Qualtrics Online Survey Platform.

### 2.4. Statistical analysis

To calculate an estimated sample size, we assumed the average score on “If given the opportunity to take the COVID-19 vaccine, how likely is it that you would get the vaccine/shot?” in the pre-survey as around 1.5 based on published COVID-19 attitude surveys as of February 2021. We expected our educational intervention would increase the average score for this question on the post-survey to be around 2.0 with the standard deviation of paired difference to be around 1. To achieve 95% power with Alpha = 0.05, we estimated the sample size of 84 to detect medium effect size differences among subgroup means. Frequency distributions were computed for each of the demographic variables. Independent two-sample *t*-test and ANOVA were used to measure participants’ attitudes and knowledge toward COVID-19 and vaccines. Normality and homogeneity of variance were checked for both ANOVA and *t*-tests. Attitude was assessed through the following variables: belief in CDC recommendations, concern about mild reactions to COVID-19 infection, trust in the vaccine, belief in adequate testing of the vaccine, concern about the vaccine being developed too quickly, concerns about side effects of the vaccine, past mistreatment with medical care, and trust of the source. Knowledge was assessed with questions pertaining to the following variables: protection & reduction of COVID-19 transmission, how COVID-19 spreads, how vaccines work, how COVID-19 vaccines work, being cautious in public, COVID-19 vaccine side effects, and COVID-19 vaccine development. Paired *t*-tests were used to measure the change in participants’ attitudes and knowledge toward COVID-19 and vaccines to compare changes between the pre-intervention survey and the post-intervention survey. An unpaired *t*-test was utilized to compare changes in the participants’ attitude and knowledge between infographic and videographic intervention. Changes associated with the demographics were analyzed *via t*-tests and ANOVA. Finally, means were calculated to compare changes between the pre-intervention, post-intervention, and 3-month follow-up questionnaires. Statistical analysis was completed *via* Statistical Package for Social Sciences (SPSS).

## 3. Results

**Figure 3:**
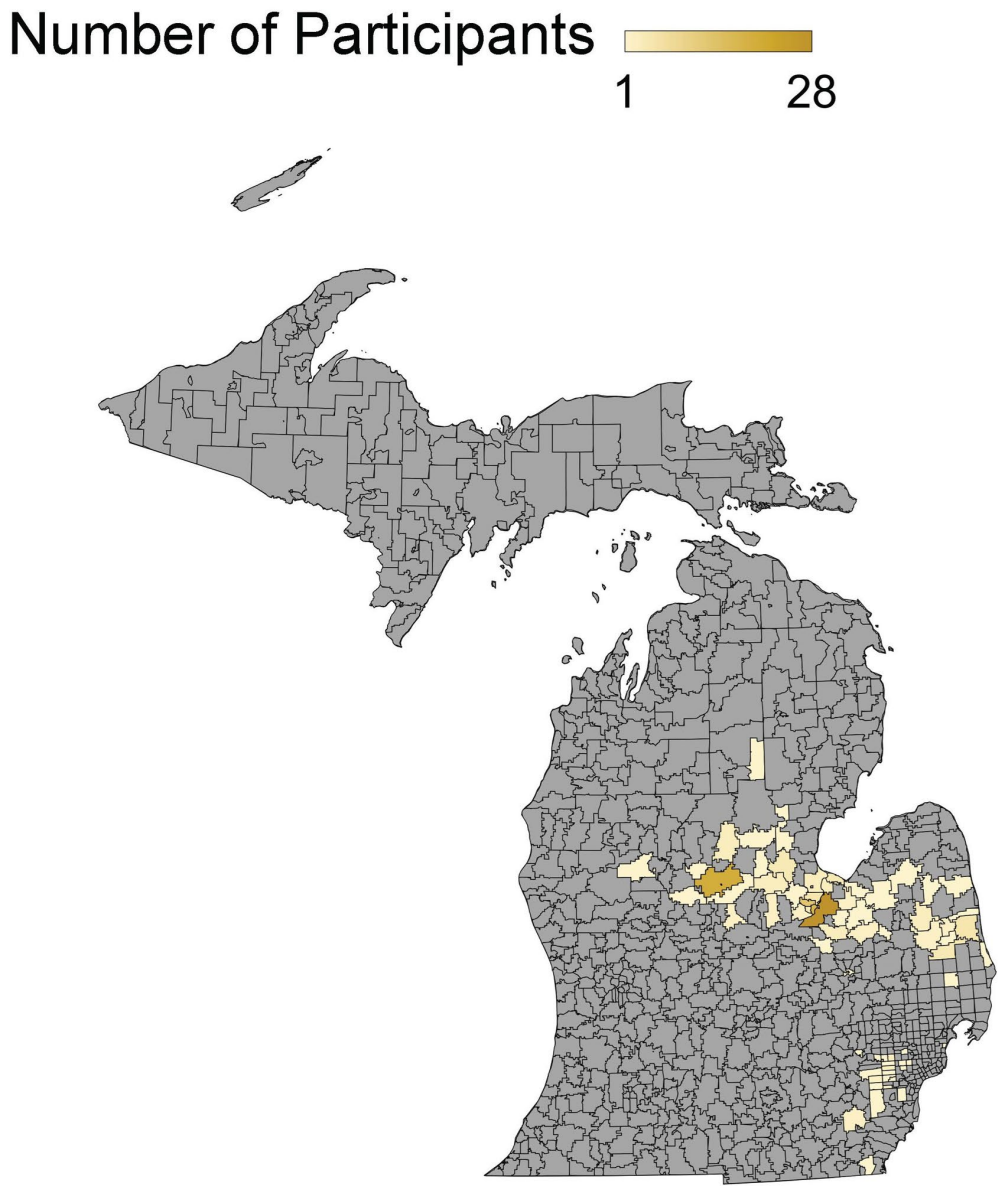
Participant population density map. Geographical areas represented among participant population.

**Figure 4:**
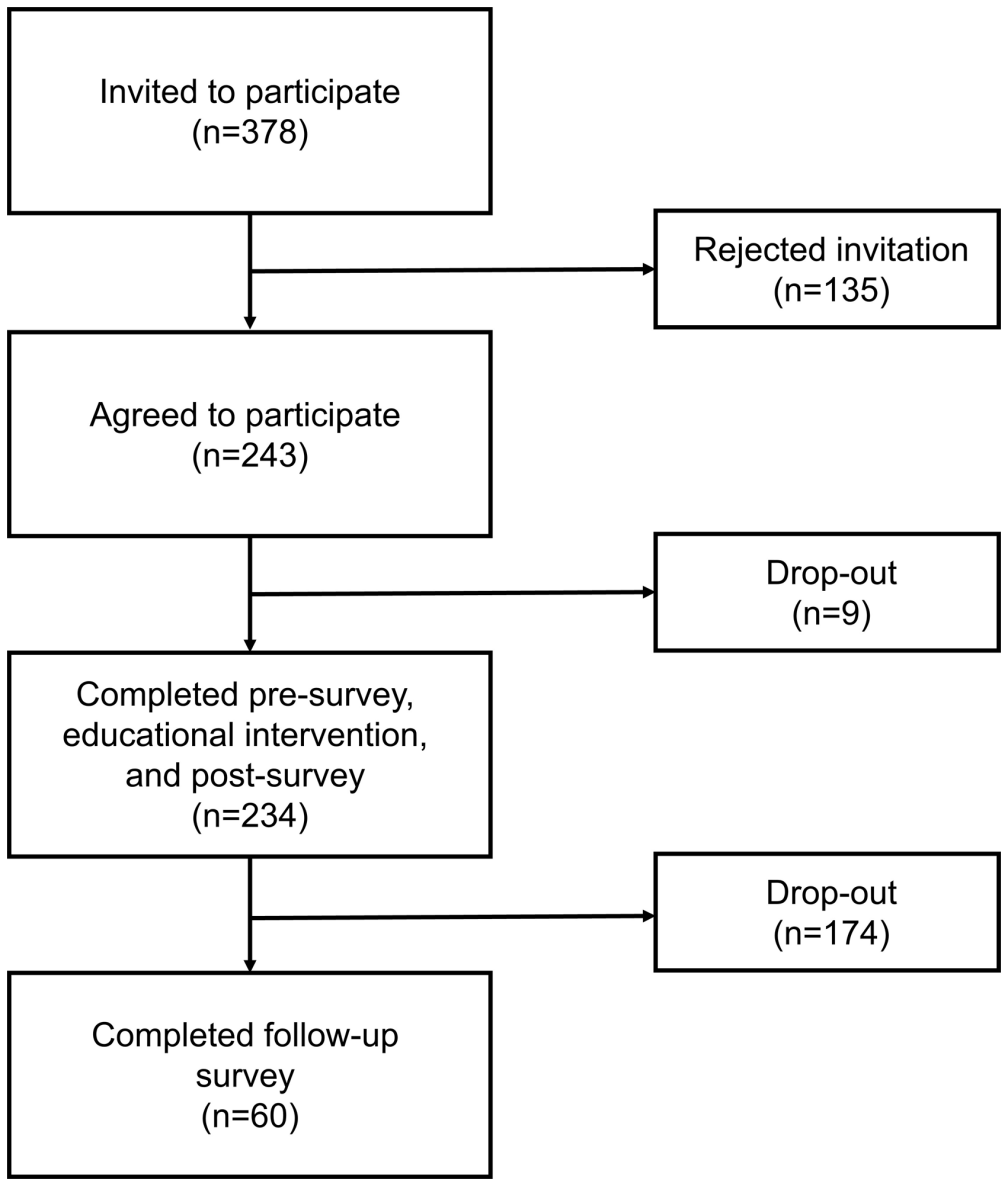
Flow diagram of response and completion rate.

234 participants, who reside in 18 counties throughout Michigan, completed the pre-survey, educational intervention, and post-survey (Figure 3). 60 patients completed the follow-up survey (Figure 4). The demographic data from the cohort of 234 participants can be seen in Table 1. The cohort was comprised of 76% females with the most common age range being 25–34 years old (31.3%). Most participants identified their ethnicity as White or Caucasian (69.3%), with those identifying as Black or African American as the second most common ethnicity (24.4%). Race was assessed and 93% identified as non-Hispanic. Additionally, most participants reported their residence as metropolitan (69.0%) with 35.6% of respondents falling into the household income bracket of $15,001–$45,000 per year. When asked about education, most participants recorded having some college credit but no degree (27%) or having a high school diploma or GED (27.1%). Political affiliations and religion were assessed with 42.9% identifying as democrat and 75% identifying as Christian. Demographic information regarding COVID-19 was also assessed with 45.6% reporting that their employment status was impacted by COVID-19 and 41.9% recorded themselves as essential workers. Of those surveyed, 71% reported testing themselves for COVID-19 in the past and 64.3% noted that they knew someone who tested positive for COVID-19 in the past. Participants were also asked whether they knew someone who was either hospitalized or who died from COVID-19 and 52.2% reported that they did know someone. The questionnaire also inquired whether participants had any underlying at-risk medical conditions (i.e., cancer, diabetes, HIV, and *etcetera*) with 38.8% responding that they had one at-risk condition and 32.0% reporting they had two or more at-risk conditions. They were also asked whether anyone in their household had at-risk medical conditions with 23.0% stating that someone in their household had one condition and 23.5% reporting someone in their household had two or more conditions. Participants were also provided with a list of CDC precautions including wearing a mask, social distancing, and washing hands often, *etcetera*, with 24.1% of people reporting following all 10 listed precautions. Lastly, participants were asked about previous vaccinations. 53.5% of participants reported receiving the influenza vaccine last year and 55.5% reported that they either had already received it or were planning to get the vaccine this year.

**Table 1:** Demographics of study cohort.

Characteristic	Total *N* (%)
Gender	
Female	158 (76%)
Male	49 (23.6%)
Other	1 (0.5%)
**Age**	
18–24 years	31 (13.7%)
25–34 years	71 (31.3%)
35–44 years	41 (18.1%)
45–54 years	19 (8.4%)
55–64 years	25 (11%)
65+ years	22 (9.7%)
Missing	18 (7.9%)
Ethnicity	
White or Caucasian	142 (69.3%)
Black or African American	50 (24.4%)
American Indian or Alaska Native	2 (1.0%)
Asian	3 (1.5%)
Other	8 (3.9%)
Race	
Hispanic	12 (7.0%)
Non-Hispanic	159 (93%)
Residence	
Metropolitan	140 (69.0%)
Non-Metropolitan	63 (31.0%)
Income	
Less than $15,000	54 (26.3%)
$15,001–45,000	73 (35.6%)
$45,001–90,000	55 (26.8%)
$90,001–150,000	16 (7.8%)
Over $150,000	7 (3.4%)
Education	
Did not finish high school	11 (5.3%)
High School diploma or GED	56 (27.1%)
Some college credit, no degree	57 (27.5%)
Trade/technical/vocational training	20 (9.7%)
2-year college degree or Associate’s	17 (8.2%)
4-year college degree or Bachelor’s	33 (15.9%)
Master’s degree	11 (5.3%)
Doctorate degree	2 (1.0%)
Political affiliation	
Republican	42 (20.5%)
Democrat	88 (42.9%)
Independent	40 (19.5%)
Something else	35 (17.1%)
Religion	
Christian	147 (75.0%)
Other religions	49 (25.0%)

As seen in Table 2, after participants completed either educational intervention, participants showed an increased positive attitude regarding COVID-19 and its vaccine. Results demonstrated that participants had increased belief in CDC recommendations (*p =* 0.005), trust in the vaccine (*p* = 0.005), agreed that the COVID-19 vaccines were adequate tested (*p =* 0.001), identified that they had experienced mistreatment in the medical care system in the past (*p =* 0.005), agreed that the source that told them to receive a COVID-19 vaccination were trustworthy (*p =* 0.015), and worried about taking time off of work to get a COVID-19 vaccine (*p =* 0.023). In addition, after the intervention, less participants were concerned about mild reactions from COVID-19 infection (*p =* 0.005), the rapid development of the vaccines (*p =* <0.001), or the side effects of a COVID-19 vaccine (*p =* 0.031). There was no statistical significance concerning the long-term side effects of the vaccine, trust in the healthcare system, the inability to find childcare to obtain the vaccine, or hesitancy to obtain the vaccine due to religious beliefs.

**Table 2:** Mean item virus and vaccine attitude difference scores (*N* = 164).

Abbreviated item	Pre	Post	Difference	Paired-*t*-test	*p* value
Belief in CDC recommendations	1.55	1.69	0.14	2.84	**0.005**
Concern about mild reaction of virus infection	1.04	0.86	−0.18	−2.86	**0.005**
Trust in the vaccine	1.26	1.44	0.18	3.29	**0.001**
Adequate testing of vaccine	1.31	1.41	0.1	2.37	**0.019**
Vaccine developed too quickly	1.01	0.85	−0.16	−3.40	**<0.001**
Concern about side effect of vaccine	1.04	0.93	−0.11	−2.18	**0.031**
Concern about long-term effects of vaccine	1.12	1.02	−0.1	−1.96	0.052
Trust in healthcare	1.54	1.61	0.07	1.78	0.077
Past mistreatment with medical care	0.56	0.67	0.11	2.83	**0.005**
Trusted source	1.29	1.42	0.13	2.47	**0.015**
Do not have time to receive vaccine	0.14	0.24	0.1	2.29	**0.023**
Cannot find childcare	0.98	0.86	−0.12	−1.67	0.097
Religion	0.11	0.17	0.06	1.68	0.095

Bold values represent statistically significant values (*p* < 0.05).

After demographic differences were assessed between participants, there was still an overall increase in positive attitude concerning the vaccine post-intervention (Supplementary Table 1). Those who identified as female were less likely to agree that the vaccine was developed too quickly following the intervention (*p* = 0.04). When examining different racial groups, those who identified as White showed increased trust in the vaccine post-intervention, whereas those who are non-White showed a decreased trust in the vaccine after the intervention (*p =* 0.04). Overall, all age groups, except for those who were 65 years and older, were less concerned about mild reactions of the virus infection. Location of residence, religion, political affiliation, income, and education did not show statistically significant differences in any of the nine attitude-related categories.

Table 3 demonstrates COVID-19 and vaccine knowledge pre- and post-educational intervention. Out of the seven knowledge-based questions, knowledge regarding COVID-19 and vaccine topics increased in six of these variables. After completing the intervention, participants showed increased knowledge in the following: protection and reduction of COVID-19 transmission (*p* = <0.001), how COVID-19 spreads (*p =* 0.026), how vaccines work (*p =* 0.005), being cautious in public (*p =* 0.019), COVID-19 vaccine side effects (*p =* 0.0047), and understanding of COVID-19 vaccine development (*p =* 0.008). The only knowledge topic that did not show significant increase post-intervention was: How the COVID-19 vaccine works (*p* = 0.18).

**Table 3:** Mean item virus and vaccine knowledge difference scores.

Abbreviated Item	Pre	Post	Difference	Paired-*t*-test	*p* value
Protection and reduction of COVID-19 transmission	1.26	1.5	0.24	4.44	**<0.001**
How COVID-19 spreads	1.64	1.74	0.1	2.24	**0.026**
How vaccines work	1.68	1.8	0.12	2.84	**0.005**
How COVID-19 vaccine works	1.7	1.76	0.06	1.35	0.18
Being cautious in public	1.5	1.64	0.14	2.36	**0.019**
COVID-19 vaccine side effects	1.61	1.7	0.09	2.00	**0.047**
COVID-19 vaccine development	1.35	1.47	0.12	2.69	**0.008**

Bold values represent statistically significant values (*p* < 0.05).

Knowledge-based questions were also stratified by demographics (Supplementary Table 2). After the intervention, all age groups, except for those who were 45–54 years old, showed improved knowledge regarding the protection and reduction of COVID-19 questions (*p =* 0.057). Individuals between 18–24 and 55 years and older showed improved knowledge regarding COVID-19 vaccine side effects (*p* = 0.045); those ages 25–34 and 45–54 showed no improvement (*p* = 0.045); those who were 35–44 years showed less knowledge in this area despite the intervention (*p* = 0.045). After the intervention, those with an income less than $15,000 showed a decrease in knowledge regarding the protection and reduction of COVID-19 transmission, while other income classes improved their knowledge after the intervention (*p =* 0.005). All political parties showed an increase in knowledge regarding protection and reduction of COVID-19 transmission (*p =* 0.018). Among the political parties, those who identified as independent showed the least improvement.

When comparing the effectiveness of the two educational intervention modalities (infographic vs. videographic), there were no statistically significant differences in either attitude or knowledge. Overall, there was a high correlation of vaccination acceptance before and after both modalities of educational intervention; however, there was no significant change in vaccine acceptance post-intervention (Supplementary Tables 3–5).

Mean values were calculated to demonstrate the participant attitude between the pre-intervention, post-intervention, and follow-up questionnaires (Table 4). Due to the reduced sample size in the post-intervention group being below the required estimated sample size threshold, repeated measures analyses across the three time points were not computed. However, for informational purposes, we included the follow-up means. There was an increase in attitude when comparing pre-intervention to the follow-up survey in the following variables: belief in CDC recommendations, trust in the vaccine, adequate testing of the vaccine, concerns of past mistreatment with medical care, and agreement that sources that told them to receive a COVID-19 vaccination were trustworthy. However, these variables also demonstrated a decrease in attitude when comparing post-intervention and the follow-up survey. Additionally, there was a decrease in the following variables when comparing the pre-intervention and follow-up questionnaire: concerns about mild reaction of virus infection, vaccine developed too quickly, and concerns about side effects of the vaccine. However, these showed an increase when comparing the post-intervention to the follow-up survey. Results from this indicate that although attitude improved when comparing pre-intervention to follow-up, the largest improvement occurred when comparing pre-intervention to post-intervention.

**Table 4:** Mean item virus and vaccine attitudes difference scores for all timepoints.

Abbreviated Item	Pre (*N* = 164)	Post (*N* = 164)	Follow-up (*N* = 60)
Belief in CDC recommendations	1.55	1.69	1.56
Concern about mild reaction of virus infection	1.04	0.86	0.78
Trust in the vaccine	1.26	1.44	1.29
Adequate testing of vaccine	1.31	1.41	1.37
Vaccine developed too quickly	1.01	0.85	0.9
Concern about side effects of vaccine	1.04	0.93	0.94
Past mistreatment with medical care	0.56	0.67	0.6
Trusted source	1.29	1.42	1.35

Knowledge was also assessed longitudinally by calculating mean values (Table 5). There was an increase in six out of the seven knowledge variables when comparing pre-intervention to the follow-up survey: protection and reduction of COVID-19 transmission, how COVID-19 spreads, how the COVID-19 vaccine works, being cautious in public, COVID-19 side effects, and COVID-19 vaccine development. Additionally, the measured items “how COVID-19 spreads” and “being cautious in public” demonstrated an increase in knowledge from post-intervention and the follow-up survey. The remaining variables showed a similar trend to that seen in attitude, with a decrease in knowledge when comparing post-intervention to follow-up. The knowledge variable of “how vaccines work” did show an increase from pre-intervention to post-intervention; however, a decrease in level of knowledge was seen at follow-up when compared to either pre- or post-intervention.

**Table 5:** Mean item virus and vaccine knowledge difference scores for all timepoints.

Abbreviated item	Pre (*N* = 164)	Post (*N* = 164)	Follow-up (*N* = 60)
Protection and reduction of COVID-19 transmission	1.26	1.5	1.29
How COVID-19 spreads	1.64	1.74	1.87
How vaccines work	1.68	1.8	1.6
How COVID-19 vaccine works	1.5	1.64	1.6
Being cautious in public	1.61	1.7	1.77
COVID-19 vaccine side effects	1.35	1.47	1.45
COVID-19 vaccine development	1.26	1.5	1.29

## 4. Discussion

### 4.1. Utilizing educational interventions to improve attitude and knowledge toward vaccination

Vaccine hesitancy has been steadily increasing over the past few decades and was declared a top 10 global health threat by the WHO in 2019 (23). Despite this increasing trend, vaccination against SARS-CoV-2 remains the most widely accepted method of protection against serious illness, hospitalization, and death (24). Reiter et al. demonstrated that individuals with a positive perception of the COVID-19 vaccine are more likely to receive the vaccine (25). In addition, improved knowledge surrounding SARS-CoV-2 and the COVID-19 vaccine has been shown to improve vaccination acceptance (26). Thus, it is imperative to improve perceptions and knowledge of the vaccine to improve overall vaccine uptake. However, the question remains of how to improve perception. A previous study by Kaim et al. determined that videographic educational interventions improved attitudes toward vaccination acceptability (20). In our study, we expand upon this to include another educational modality, infographic, to determine if there is a difference in attitude or knowledge surrounding SARS-CoV-2 or the COVID-19 vaccine based on the two different educational modalities. We also evaluate whether the effects of these educational interventions wain over time.

This study found that both modalities of educational intervention improved overall attitudes in nine out of the 13 variables assessed. Post-intervention, there was an overall increased trust in the vaccine with increased belief that the vaccines were adequately tested and were not developed too quickly. There was also less concern regarding mild reactions or side effects from the COVID-19 vaccine. After the intervention, there was an overall increase in participants’ recognition that of previous medical mistreatment; however, there was also an increase in participants agreement that sources (i.e., media, government institutions, and *etcetera*) encouraging them to receive a COVID-19 vaccination were trustworthy. The findings also identified that there was an increased concern that their employment or schedule will not permit time off work to obtain a vaccine. Therefore, it may be of benefit for policymakers to incentivize employers to allow workers time off to obtain the vaccine, as well as time off for any side effects from the vaccine.

The study also found that both modalities of educational intervention improved knowledge in six out of the seven knowledge-based variables. Post-intervention, there was an increase in knowledge regarding how COVID-19 spreads and how to protect against and reduce the transmission of COVID-19. Participants also showed increased knowledge regarding precautions to take in public to reduce the risk of COVID-19 infection. Additionally, there was an increased knowledge regarding COVID-19 vaccine side effects and understanding of the COVID-19 vaccine development. Previous studies have demonstrated that higher levels of knowledge correlate with higher levels of vaccine acceptance (27, 28). Data from this study support these previous findings along with those of Kaim et al. with regard to educational interventions improving vaccine acceptance (20). In addition, Kreps et al. identified that vaccinations that were endorsed by CDC and WHO were associated with higher vaccination acceptance (29). Therefore, utilizing information from CDC and WHO, as done in this study, may be a useful approach in increasing vaccination acceptance.

While both educational modalities demonstrated significant increases in attitude and knowledge regarding COVID-19 and vaccines, there was not a significant difference between the infographic versus videographic intervention when comparing the assessed variables. A previous study found that both educational handouts and educational videos improved knowledge scores and acceptability of the HPV vaccine; however, educational videos were associated with higher levels of knowledge and acceptability (30). While the previous study demonstrated videographic representation of information was associated with greater levels of knowledge and acceptability, there is still limited research on the effectiveness of different modalities of educational intervention on vaccine hesitancy (19). Although our study aimed to further this understanding, there was no statistical significance noted between the two forms of educational intervention. This may be due to the ceiling effect as most participants reported high levels of vaccine acceptance both pre- and post-intervention. Therefore, further investigations are needed to determine the optimal format of educational intervention. Based on our findings and limited prior research, we propose that public health officials utilize the most practical (i.e., cost-efficient, easily dispersible, and *etcetera*) educational intervention until further studies determine the most effective educational modality.

### 4.2. Demographics that need more attention during interventions

While this study demonstrated an overall improvement in attitudes and knowledge toward the COVID-19 post-intervention, it is important to consider the demographics of participants. This study utilized the HBM as a theoretical framework to examine variables that impact vaccine hesitancy. A previous systematic review examined the influence of HBM constructs on COVID-19 vaccine hesitancy and found that gender, education, age, geographical location, occupation, income, employment, marital status, race, and ethnicity were all associated with vaccine hesitancy (18). We examined many of these variables as well as others as seen in Table 1. The study found that those who identified as female were less likely to agree that the vaccine was developed too quickly post-intervention compared to those who identified as men; however, there was no statistical significance between genders on other measured attitude variables. Interestingly, a previous study demonstrated that women were more likely to say they were unsure to take the COVID-19 vaccine when available (31). This study cited concerns about personal health, such as potential side effects, as a potential reason for this gender discrepancy. Our study did not find that females were concerned about potential side effects. This may be explained by the fact that the study performed by Prickett et al. collected data in March of 2021, when less was known about SARS-CoV-2 and the COVID-19 vaccines. It is also possible that geographic differences or the larger sample size of Prickett et al. played a role in the differences.

The study also demonstrated that those identifying as White showed increased trust in the vaccine while those who identified as non-White, which was mainly comprised of those identifying as Black or African American, showed a decreased trust in the vaccine post-intervention. This is consistent with previous studies demonstrating that Black or African American populations have greater mistrust in government institutions and greater vaccine hesitancy when compared to White populations (32–34). While reasons behind this are multifactorial, history shows that unethical research such as the Tuskegee Syphilis Study plays a significant role in distrust in medical institutions and vaccine hesitancy (35). Therefore, it is imperative to recognize past injustices and continue to improve upon these ethnic disparities through increased transparency, education, and accessibility regarding public health initiatives in minority communities.

Regarding age, there were significant differences noted among ages concerning attitude and knowledge. Overall, all age groups, except for those who were 65 years and older, were less concerned about mild reactions to the virus infection post-intervention. Both modalities of intervention utilized information gathered from the WHO and CDC, which emphasized the morbidity and mortality associated with increased age and COVID-19 infection (21, 22). This likely explains the trends seen regarding this variable. Regarding knowledge, those in the age range between 25–34 and 45–54 showed no improvement post-intervention regarding COVID-19 vaccine side effects. Those in the age range 35–44 showed decreased knowledge in this variable post-intervention. Interestingly, Gravelle et al. reported that individuals aged 25–49 were the group most hesitant towards vaccination and associated this age range with those who are most likely to be parents (36). In addition, their study found that those aged 50–64 broadly supported vaccines, but still had concerns. The age range reported in our study falls between these two age ranges and, thus, our findings may reflect the possible reasons for hesitancy suggested by Gravelle et al. The 25–54 age range may be more likely to be parents and have additional concerns related to parenthood and children that the educational intervention did not address. Moreover, a previous study demonstrated that concern regarding COVID-19 vaccine side effects was a significant factor that increased vaccine hesitancy (37). Thus, information targeted toward the age ranges of 25–54, specifically information targeted toward parents, may improve vaccine acceptance.

In addition, the study found that those in the lowest household income bracket showed a decrease in knowledge regarding the protection and reduction of COVID-19 transmission, while other variables were not statistically significant. This finding is supported by the work of Latkin et al., which found that income was an independent predictor of reduced vaccine uptake and increased hesitancy (38). Latkin et al. also demonstrated that political conservatism was associated with reduced vaccine uptake and hesitancy. Our results did not support this as six out of the seven measured variables were not statistically significant in relation to political affiliation. The only statistically significant measured variable was protection and reduction of COVID-19 transmission, with those identifying as republican showing greater knowledge post-intervention compared to those identifying as democrat or independent. This may be due to sampling bias as nearly double the percentage of participants identified as democrat versus republican. Regardless, income did show a decrease in knowledge indicating that lower socioeconomic groups may benefit from targeted information to improve vaccine acceptance.

### 4.3. The role of educational interventions in the short-term and long-term to address public health issues and the participatory, action, and research cycle model to address public health issues

Despite slight variation in demographics, findings demonstrated that overall, there was an increase in both attitude and knowledge regarding assessed variables immediately following intervention. While these results are encouraging for decreasing vaccine hesitancy in the short-term, Eitze et al. found that immediate increases in knowledge and risk perceptions of pneumococcal and influenza did not decrease vaccine hesitancy in the long-term (39). Our study attempted to test this by analyzing the changes in attitude regarding COVID-19 and the vaccine over different time periods. Out of the eight attitude-related variables assessed across pre-intervention, post-intervention, and follow-up, all eight demonstrated improvement when comparing pre-intervention to follow-up. However, there was a decrease in attitude across all eight assessed variables when comparing post-intervention and follow-up. The same was true in six out of the seven knowledge-tested variables with improved knowledge between pre-intervention and follow-up and worsening knowledge between post-intervention and follow-up. There was an increase in knowledge in the variable “how vaccines work” from pre-intervention to post-intervention; however, knowledge in the follow-up was lower than pre-intervention. These results indicate that a one-time educational intervention does improve attitudes and knowledge long-term; however, the effects of the intervention wains over time.

**Figure 5:**
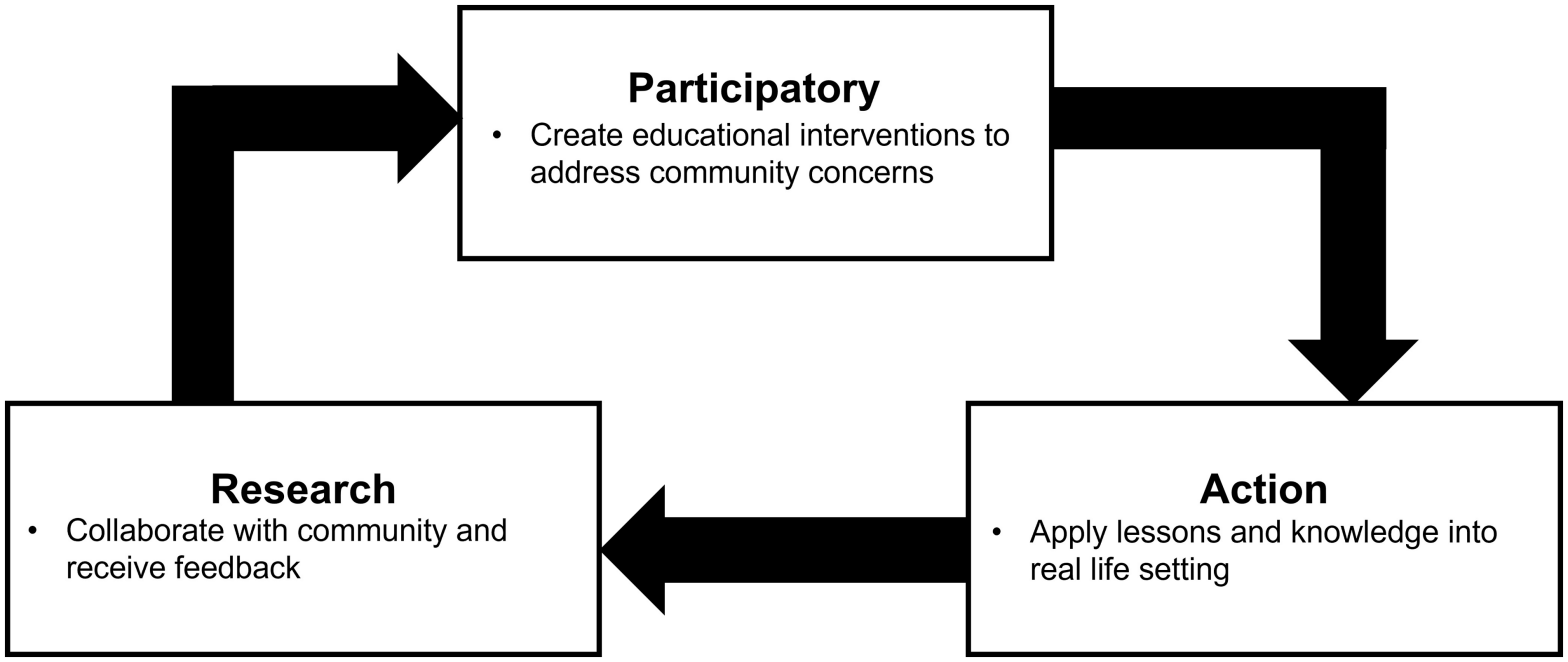
Proposal to modify the Participatory, Action, and Research (PAR) to the Participatory, Action, and Research Cycle (PARC) Model.

The decrease in attitude and knowledge over time seen in this study emphasizes the importance of utilizing educational interventions as a continuous process rather than a singular event. Therefore, we propose utilizing a modified version of the Participatory, Action, and Research (PAR) model to address vaccine hesitancy. PAR is an approach to research that emphasizes active participation by members in the target population (40). Action is achieved through analysis and reflection of data collected from community members to determine follow-up interventions. The PAR approach is rising in health research, and a recent study demonstrated that utilizing this method significantly increased vaccination rates in unvaccinated children (41). We further recommend that the PAR model be modified to the Participatory Action Research Cycle (PARC), which serves as an additional tool to create actionable plans and empower community members similar to the original PAR model (Figure 5). This model is further refined to incorporate the importance of ongoing education when addressing public health concerns, especially conceptually difficult concepts such as vaccine hesitancy.

The PARC model proposes a three-item cycle. The first is to participate in the community and to assess the attitude and knowledge of the community regarding a specific public health issue, such as vaccine hesitancy. During this part of the cycle, community members participate in an educational intervention, such as watching a video or reading an infographic. The following step is action. Community members will then apply what they have learned to make informed decisions about their body, such as receiving or not receiving a vaccine. The next step is research. During this step, researchers will reassess the participant’s knowledge and determine if there are gaps in knowledge. Gaps in knowledge are addressed and applied back during the next iteration of the cycle beginning with the participatory aspect. Reinforcing and solidifying knowledge through this active and reflective process gives the community members autonomy to use this newfound knowledge to determine what health decision is best for them.

### 4.4. Limitations

While this study has many strengths, we acknowledge that the logistical obstacles of this study led to the data collection timeline to be spread out. During this time, many pandemic-related factors, such as the various new COVID-19 strain variants, CDC recommendation changes, COVID-19 case surges, and booster shots, may have impacted participants’ opinions depending on when the participant took the survey. In addition, the limited sample may not have captured all meaningful trends. Further studies using a larger sample size could validate the demographic relationships and associations found in this study. Moreover, the sample population was derived from patients at outpatient primary care offices. This can lead to selection bias as this population may vary from the general population. Many participants also failed to record demographic information, which may have skewed demographic trends in attitude and knowledge. The cash incentive for completing these surveys may have also impacted the study as those in lower income brackets may have been overrepresented when compared to upper-income brackets. Additional limitations of this study include the requirement of participants to be literate in English and the surveys were distributed at clinics selected from convenience sampling, thereby excluding non-English speaking populations and patients from other locations, respectively. Lastly, this study also required participants to be competent in technology use, as it required an iPad or Smartphone device. Nevertheless, we believe that this study addressed many of the gaps in previous studies including how the effects of intervention wain over time and comparing different modalities of educational intervention.

### 4.5. Conclusion

Educational interventions play a key role in addressing public health issues, such as vaccine hesitancy. Effective interventions require careful planning and execution to achieve desirable changes. Our study shows the short- and long-term changes of brief educational interventions and variations in responses among demographic groups.

In addition, our study illustrated many meaningful trends for future investigation. For instance, our study demonstrated that there was not a significant difference between infographic vs. videographic educational interventions regarding improvements in attitude or knowledge; however, there are many more modalities that could be investigated. Interactive educational modalities could be included as this may improve acquisition and retention of knowledge. Future efforts could also be implemented at different follow-up time periods instead of one follow-up. This may be beneficial to determine when it would be most beneficial to implement the next cycle of educational intervention.

## Data availability statement

The original contributions presented in the study are included in the article/Supplementary material, further inquiries can be directed to the corresponding author.

## Author contributions

MT and NR devised the project and the main conceptual ideas and designed the survey. MT and NH designed the educational intervention. SH, NH, and BN revised the survey. MT, SH, AK, KG, ZS, and JH collected data. SZ performed the analytic calculations. MT assisted with the analysis and prepared the figures. MT, SZ, and ZS prepared the tables. MT and ZS were the major contributors in writing the manuscript. All authors contributed to the article and approved the submitted version.

## Funding

This work was supported in part by Michigan State Medical Society Foundation (Grant Number: P64840).

## Conflict of interest

The authors declare that the research was conducted in the absence of any commercial or financial relationships that could be construed as a potential conflict of interest.

## Publisher’s note

All claims expressed in this article are solely those of the authors and do not necessarily represent those of their affiliated organizations, or those of the publisher, the editors and the reviewers. Any product that may be evaluated in this article, or claim that may be made by its manufacturer, is not guaranteed or endorsed by the publisher.
