# Supplementary material for: The impact of educational interventions on COVID-19 and vaccination attitudes among patients in Michigan: A prospective study
Source: Front Public Health. 2023 Apr 3;11:1144659. doi: 10.3389/fpubh.2023.1144659 (PMC10106744; doi:10.3389/fpubh.2023.1144659)
Supplement: Supplementary file 1 [file Data_Sheet_1.PDF]

COVID-19 VIRUS & VACCINES

Information consulted with Dr. Nicholas Haddad, infectious disease specialist.

How do vaccines work?

Vaccines work by preparing our bodies to counteract the effect of invading organisms (pathogens).

What are the current COVID-19 vaccines & How do they differ?

These COVID-19 vaccines have been authorized for Emergency Use by the FDA as of July 2021.

Messenger RNA vaccines
(Pfizer and Moderna)
- Require two doses (shots).
- Efficacious two weeks after the second dose.

Vector subunit vaccine
(Johnson & Johnson Janssen)
- Requires one dose.
- Efficacious two weeks after that one dose.

None of these vaccines will give you the infection.
You will never get COVID-19 from a COVID-19 vaccine.

What are key things to know about the COVID-19 vaccines?

The COVID-19 vaccines are safe and effective. There have been no cutting corners in the process of development of the vaccines.

Most side effects of the vaccine are mild.
Most side effects last no more than 24 – 48 hours.
- Minor injection site reactions (pain, tenderness, redness)
- Fever or chills
- Minor muscle aches and pains
- Major common side effect is fatigue

Most side effects respond to common analgesics, such as Tylenol (acetaminophen), Advil (ibuprofen).

What are the benefits of a COVID-19 vaccine?

Protects you from getting the COVID-19 disease.

Prevents severe disease.

Eliminates risk of hospitalization and death.

Once you are fully vaccinated, you can start doing more.
such as gathering indoors with others who are fully vaccinated.

Be careful in public because others' vaccination status is unknown.
We still need to be careful of others if we are in a public place because we are not sure if they have been fully vaccinated or if they are mildly symptomatic. They can still transmit the disease.

What are we still learning about the COVID-19 vaccines?

If the vaccine will prevent us from spreading the virus to others even if we are not symptomatic at that time.

How long immunity will last.
How long the vaccine will protect people from the disease itself.

If we need a booster shot.

Effectiveness against COVID-19 variants.

The vaccines will continue to be monitored for long-term safety and efficacy.

Myths vs. Facts

FALSE — People should wear masks while exercising.

FALSE — The prolonged use of medical masks (when properly worn) causes CO2 intoxication and oxygen deficiency.
"[The masks] are never tight enough to cause [intoxication]."

TRUE — Most people who get COVID-19 recover from it.

FALSE — Catching COVID-19 means you will have it for life.
"You will develop immunity once you recover, but [the infection] goes away. [COVID-19] does not last in your body for more than 10 days."

FALSE — The COVID-19 vaccines contains magnetic material and/or includes a microchip that the government can use to track me.

FALSE — The COVID-19 vaccines cause infertility.
"There are no concerns on [the vaccines] causing infertility, neither in men or women. Additionally, there is no evidence that it has caused or concerns about it causing birth defects in pregnant women."

Which of the following may protect you from or reduce the risk of transmission of COVID-19?

DOES NOT protect you from or reduce risk of transmission of COVID-19:
- Taking vitamin and mineral supplements.
- Treatment with hydroxychloroquine.
- Treatment with dexamethasone.
- Drinking alcohol.
- Adding pepper or eating spicy foods.
- Spraying a disinfectant on your body.
- Drinking bleach.
- Exposing self to sun or high temperatures (greater than 77°F/25°C).
- Taking a hot bath.
- Hand dryers.
- Vaccination against pneumonia.
- Rinsing your nose with saline.
- Eating garlic.
- Antibiotics.

DOES protect you from or reduce risk of transmission of COVID-19:
- Handwashing.
- Wearing a mask that covers your nose, mouth, and chin.

Which of the following ways is COVID-19 spread?

DOES NOT spread:
- Water or swimming.
- Through houseflies.
- Through mosquito bites.
- Through 5G mobile networks.
- Through hot and humid climates.

DOES spread:
- Between people less than 6 feet of one another.
- Through respiratory droplets when an infected person coughs, sneezes, or talks.
- By touching a contaminated surface and then touching your eyes, nose, or mouth before washing your hands.

Where can I go to get a vaccine?

Visit Vaccines.gov

Local pharmacy

Local health department

Local news outlets

Should I get vaccinated?

YES

- Discuss with your doctor if you have concerns about the vaccine.
- Especially recommended for patients with certain risk factors, such as obesity, diabetes, high blood pressure, or patients who smoke.
- Vaccination is a good idea after recovering from COVID-19 because the vaccine can improve immunity.
- Highly encouraged for pregnant women as well.

The federal government is providing the vaccine free of charge to all people living in the United States, regardless of immigration or health insurance status.

Learn more at CDC.gov
Check out https://www.cdc.gov/coronavirus/2019-ncov/vaccines/ to learn more about the vaccines.

The COVID-19 vaccines are excellent tools to stop the pandemic and return to normal life.
