# Supplementary material for: The impact of educational interventions on COVID-19 and vaccination attitudes among patients in Michigan: A prospective study
Source: Front Public Health. 2023 Apr 3;11:1144659. doi: 10.3389/fpubh.2023.1144659 (PMC10106744; doi:10.3389/fpubh.2023.1144659)
Supplement: Supplementary file 2 [file Data_Sheet_2.docx]

|  | **Gender** | | | | **Race** | | | | **Metro** | | | | **Religion** | | | | **Age** | | | | | | | | **Political affiliation** | | | | | | | **Income** | | | | | | | **Education** | | | | | | | | |
| --- | --- | --- | --- | --- | --- | --- | --- | --- | --- | --- | --- | --- | --- | --- | --- | --- | --- | --- | --- | --- | --- | --- | --- | --- | --- | --- | --- | --- | --- | --- | --- | --- | --- | --- | --- | --- | --- | --- | --- | --- | --- | --- | --- | --- | --- | --- | --- |
| **Abbreviated Item Content** | **Female** | **Male** | **t-test** | **P-Value** | **White** | **Non-White** | **t-test** | **P-Value** | **Metro** | **Non-Metro** | **t-test** | **P-Value** | **Christian** | **Non-Christian** | **t-test** | **P-Value** | **18-24** | **25-34** | **35-44** | **45-54** | **55-64** | **65+** | **F** | **P-Value** | | **Republican** | **Democrat** | **Independent** | **Something else** | **F** | **P-Value** | | **<$15,000** | **$15,001 to $45,000** | **$45,001 to $90,000** | **$90,001 to $150,000+** | **F** | **P-value** | **Did not finish high school** | **High school diploma or GED** | **Some college credit, no degree** | **Trade/technical/vocational training** | **2-year college degree or Associate’s** | **4-year college degree or Bachelor’s** | **Advanced degree (Master's, Doctorate)** | **F** | **P-value** |
| Belief in CDC recommendations | 0.17 | 0.08 | 0.91 | 0.36 | 0.16 | 0.03 | 0.99 | 0.33 | 0.10 | 0.20 | -0.90 | 0.37 | 0.20 | 0.05 | 1.40 | 0.17 | 0.36 | 0.12 | 0.09 | 0.06 | 0.11 | 0.12 | 0.67 | 0.65 | | 0.21 | 0.06 | 0.21 | 0.13 | 0.59 | 0.62 | | 0.1364 | 0.1379 | 0.2245 | 0 | 0.497 | 0.685 | 0.1111 | 0.1739 | 0.1591 | 0.1111 | 0.1429 | 0.1429 | 0.0909 | 0.039 | 1 |
| Concern about mild reaction of virus infection | -0.17 | -0.21 | 0.27 | 0.79 | -0.21 | -0.11 | -0.64 | 0.53 | -0.13 | -0.31 | 1.43 | 0.16 | 0.20 | 0.05 | 1.18 | 0.24 | -0.61 | -0.02 | -0.13 | -0.33 | -0.06 | 0.00 | 2.67 | 0.02 | | 0.00 | -0.18 | -0.30 | -0.31 | 1.19 | 0.32 | | 0.1163 | 0.2069 | 0.1875 | 0.2353 | 0.178 | 0.911 | 0.1111 | 0.0682 | 0.186 | 0.5556 | 0.2857 | 0.1429 | 0 | 1.245 | 0.286 |
| Trust the vaccine | 0.20 | 0.10 | 1.07 | 0.29 | 0.23 | -0.09 | 2.11 | 0.04 | 0.14 | 0.31 | -1.52 | 0.13 | 0.18 | 0.15 | 0.23 | 0.82 | 0.04 | 0.18 | 0.28 | 0.17 | 0.06 | 0.40 | 0.77 | 0.58 | | 0.31 | 0.03 | 0.15 | 0.41 | 2.51 | 0.06 | | 0.2619 | 0.0345 | 0.0833 | 0 | 1.61 | 0.189 | 0.2222 | 0.1364 | 0.0976 | 0.0556 | -0.2143 | 0.2222 | 0.0909 | 1.07 | 0.383 |
| Adequate testing of vaccine | 0.14 | 0.00 | 1.81 | 0.07 | 0.10 | 0.12 | -0.22 | 0.83 | 0.15 | 0.04 | 1.07 | 0.29 | 0.12 | 0.03 | 1.10 | 0.27 | 0.30 | 0.07 | 0.16 | -0.06 | -0.06 | 0.14 | 1.33 | 0.25 | | 0.22 | 0.09 | 0.03 | 0.07 | 0.75 | 0.53 | | -0.2791 | -0.1786 | -0.0851 | -0.0625 | 0.889 | 0.448 | -0.375 | -0.1395 | -0.3571 | -0.1111 | -0.2143 | 0 | 0.1818 | 1.813 | 0.1 |
| Vaccine developed too quickly | -0.22 | 0.00 | -2.06 | 0.04 | -0.17 | -0.11 | -0.49 | 0.63 | -0.15 | -0.21 | 0.61 | 0.54 | -0.18 | -0.03 | -1.58 | 0.12 | 0.04 | -0.17 | -0.25 | -0.11 | -0.33 | -0.21 | 1.00 | 0.42 | | -0.14 | -0.05 | -0.23 | -0.40 | 2.37 | 0.07 | | -0.2326 | 0 | -0.1224 | -0.125 | 1.084 | 0.358 | -0.3333 | -0.2558 | -0.0233 | -0.1111 | 0.0714 | -0.0357 | -0.0909 | 0.929 | 0.476 |
| Concern about side effect of vaccine | -0.12 | -0.08 | -0.42 | 0.68 | -0.14 | 0.06 | -1.59 | 0.12 | -0.06 | -0.18 | 1.30 | 0.20 | -0.08 | -0.23 | 1.33 | 0.19 | -0.21 | -0.07 | -0.22 | 0.11 | 0.06 | -0.27 | 1.22 | 0.30 | | -0.25 | 0.03 | -0.12 | -0.24 | 2.04 | 0.11 | | 0.0732 | 0.0714 | 0.2083 | 0.125 | 0.707 | 0.549 | 0 | 0.0476 | 0.1905 | 0.2778 | 0.1429 | 0 | 0.2 | 0.874 | 0.515 |
| Past mistreatment with medical care | 0.14 | 0.05 | 0.86 | 0.39 | 0.09 | 0.21 | -1.08 | 0.29 | 0.14 | 0.07 | 0.74 | 0.46 | 0.16 | 0.03 | 1.87 | 0.06 | 0.04 | 0.15 | 0.03 | 0.33 | 0.00 | 0.21 | 1.19 | 0.32 | | 0.17 | 0.11 | 0.06 | 0.14 | 0.27 | 0.85 | | 0.093 | 0.1754 | 0.1224 | 0.125 | 0.121 | 0.948 | 0.2222 | 0.1395 | 0.2558 | -0.1111 | 0.0714 | 0.1429 | 0 | 0.703 | 0.647 |
| Received vaccine because of trusted source | 0.11 | 0.20 | -0.70 | 0.49 | 0.18 | -0.06 | 1.79 | 0.08 | 0.14 | 0.13 | 0.09 | 0.93 | 0.09 | 0.25 | -1.43 | 0.16 | 0.18 | 0.25 | -0.06 | 0.11 | 0.22 | -0.07 | 1.19 | 0.32 | | 0.22 | 0.12 | 0.12 | 0.07 | 0.28 | 0.84 | | 0.2439 | 0.0179 | 0.102 | 0 | 1.553 | 0.203 | 0.2222 | -0.1163 | 0.25 | 0.2222 | 0.0714 | 0 | 0.3636 | 2.579 | 0.021 |
| Don't have time to receive vaccine | 0.13 | 0.00 | 1.60 | 0.11 | 0.06 | 0.19 | -0.92 | 0.37 | 0.14 | 0.02 | 1.74 | 0.08 | 0.12 | 0.08 | 0.66 | 0.51 | 0.00 | 0.22 | 0.03 | 0.19 | 0.00 | 0.00 | 1.14 | 0.34 | | 0.08 | 0.17 | 0.03 | 0.03 | 0.68 | 0.57 | | -0.3095 | -0.1404 | -0.0612 | -0.2941 | 0.913 | 0.436 | -0.4444 | -0.1905 | -0.2045 | -0.2222 | -0.0714 | -0.0357 | -0.1818 | 0.379 | 0.892 |

**Supplemental Table 1.** Mean Item Virus and Vaccine Attitudes Difference Scores by Demographic Groups

|  | **Age** | | | | | | | | | **Income** | | | | | | | **Education** | | | | | | | | | **Politics** | | | | | |
| --- | --- | --- | --- | --- | --- | --- | --- | --- | --- | --- | --- | --- | --- | --- | --- | --- | --- | --- | --- | --- | --- | --- | --- | --- | --- | --- | --- | --- | --- | --- | --- |
| **Abbreviated Item** | **18-24** | **25-34** | **35-44** | **45-54** | **55-64** | **65+** | **F** | **P-value** | **<$15,000** | | **$15,001 to $45,000** | **$45,001 to $90,000** | **$90,001 to $150,000+** | **F** | **P-value** | **Did not finish high school** | | **High school diploma or GED** | **Some college credit, no degree** | **Trade/technical/vocational training** | **2-year college degree or Associate’s** | **4-year college degree or Bachelor’s** | **Advanced degree (Master's, Doctorate)** | **F** | **P-value** | **Republican** | **Democrat** | **Independent** | **Something else** | **F** | **P-value** |
| Protection & reduction of COVID-19 transmission | 0.08 | 0.3333 | 0.4138 | -0.1875 | 0.2667 | 0.2941 | 2.199 | 0.057 | -0.0714 | | 0.3684 | 0.381 | 0.2143 | 4.446 | 0.005 | 0.1111 | | 0.2045 | 0.25 | 0.125 | 0.4286 | 0.3913 | 0.1 | 0.572 | 0.752 | 0.3243 | 0.1333 | 0.0345 | 0.5357 | 3.444 | 0.018 |
| How COVID-19 spreads | 0.1724 | 0.0175 | 0.1176 | 0.0526 | 0.0526 | 0.3 | 0.787 | 0.56 | -0.087 | | 0.1333 | 0.2 | 0.2 | 2.203 | 0.09 | 0.2436 | | -0.0909 | 0.1489 | 0.1064 | -0.0556 | 0.0714 | 0.2414 | 0.758 | 0.604 | 0.2632 | 0.0411 | 0.0882 | 0.0667 | 1.18 | 0.319 |
| How vaccines work | 0.1379 | 0.0351 | 0.0313 | 0.3333 | 0.2778 | 0.1579 | 1.252 | 0.287 | 0.0682 | | 0.1695 | 0.14 | 0.0556 | 0.367 | 0.777 | 0.3333 | | 0.1957 | 0.1489 | -0.1111 | 0 | 0.0714 | 0.1818 | 1.046 | 0.398 | 0.2432 | 0.0704 | 0.1515 | 0.069 | 0.871 | 0.458 |
| Being cautious in public | 0.2069 | 0.2679 | 0.1212 | -0.0588 | 0 | 0.0556 | 0.666 | 0.65 | 0.0698 | | 0.0678 | 0.2708 | 0.2632 | 0.812 | 0.489 | 0.2222 | | 0.0435 | 0.0667 | 0.0556 | 0.3571 | 0.4138 | 0 | 0.988 | 0.435 | 0.1944 | 0.0563 | 0.0606 | 0.3571 | 1.102 | 0.35 |
| COVID-19 vaccine side effects | 0.2069 | 0 | -0.0303 | 0 | 0.4118 | 0.1053 | 2.327 | 0.045 | 0.0732 | | 0.0678 | 0.16 | -0.0526 | 0.79 | 0.501 | 0.1111 | | 0.1556 | 0.1304 | -0.1765 | 0.0714 | 0.069 | 0 | 0.911 | 0.489 | 0.1667 | 0.0145 | 0.0882 | 0.1379 | 0.787 | 0.503 |
| COVID-19 vaccine development | 0.069 | 0.1404 | 0.125 | 0.0556 | 0.2778 | 0.0526 | 0.396 | 0.851 | 0.0455 | | 0.1552 | 0.1 | 0.3158 | 1.007 | 0.391 | 0.125 | | 0.0652 | 0.0638 | 0.1667 | 0.2857 | 0.2069 | 0.0909 | 0.436 | 0.854 | 0.2703 | 0.0714 | 0.0294 | 0.2069 | 1.423 | 0.238 |

**Supplemental Table 2**. Mean Item Virus and Vaccine Knowledge Difference Scores by Demographic Groups

**Supplemental Table 3**. Mean Item COVID-19 Vaccine Acceptance Difference Scores

| **Abbreviated Item** | **Pre** | **Post** | **Difference** | **Paired-t-test** | **P-value** |
| --- | --- | --- | --- | --- | --- |
| Likelihood of receiving vaccine | 5.6 | 5.56 | -0.04 | -0.59 | 0.558 |
| COVID-19 concern | 2.35 | 2.35 | 0 | 0 | 1 |

**Supplemental Table 4.** Mean Item COVID-19 Vaccine Acceptance Difference Scores Vaccinated Grouped

| **Abbreviated Item** | **Pre** | **Post** | **Difference** | **Paired-t-test** | **P-value** |
| --- | --- | --- | --- | --- | --- |
| Likelihood of receiving vaccine | 5.0455 | 5.0284 | -0.0171 | 0.37 | 0.740 |

**Supplemental Table 5.** Mean Item COVID-19 Vaccine Acceptance Difference Scores Likert Scale Only

| **Abbreviated Item** | **Pre** | **Post** | **Difference** | **Paired-t-test** | **P-value** |
| --- | --- | --- | --- | --- | --- |
| Likelihood of receiving vaccine | 5.0455 | 5.0284 | 0.12 | 1.32 | 0.191 |
